# Gut microbiota associate with disease severity and survival in idiopathic pulmonary fibrosis

**DOI:** 10.1093/ajrccm/aamag249

**Published:** 2026-05-19

**Authors:** John S Kim, Abigail Loe, Shwu-Fan Ma, Piyush Ranjan, Jay H Lipinski, Sheridan G Mikhail, Stephen J Gurczynski, Xiaofeng Zhou, Gary B Huffnagle, John Erb Downward, Joseph D Metcalf, Nicole Falkowski, Kathleen A Stringer, Robert P Dickson, Yong Huang, Bethany B Moore, Fernando J Martinez, Susan Murray, Imre Noth, David N O’Dwyer

**Affiliations:** Department of Medicine, University of Virginia School of Medicine, Charlottesville, VA, United States; Department of Biostatistics, University of Michigan School of Public Health, Ann Arbor, MI, United States; Department of Medicine, University of Virginia School of Medicine, Charlottesville, VA, United States; Division of Pulmonary and Critical Care Medicine, Department of Medicine, University of Michigan Medical School, Ann Arbor, MI, United States; Division of Pulmonary and Critical Care Medicine, Department of Medicine, University of Michigan Medical School, Ann Arbor, MI, United States; Division of Pulmonary and Critical Care Medicine, Department of Medicine, University of Michigan Medical School, Ann Arbor, MI, United States; Department of Microbiology and Immunology, University of Michigan Medical School, Ann Arbor, MI, United States; Department of Microbiology and Immunology, University of Michigan Medical School, Ann Arbor, MI, United States; Division of Pulmonary and Critical Care Medicine, Department of Medicine, University of Michigan Medical School, Ann Arbor, MI, United States; Department of Microbiology and Immunology, University of Michigan Medical School, Ann Arbor, MI, United States; Department of Molecular, Cellular and Developmental Biology, University of Michigan Medical School, Ann Arbor, MI, United States; Department of Microbiology and Immunology, University of Michigan Medical School, Ann Arbor, MI, United States; Department of Microbiology and Immunology, University of Michigan Medical School, Ann Arbor, MI, United States; Division of Pulmonary and Critical Care Medicine, Department of Medicine, University of Michigan Medical School, Ann Arbor, MI, United States; Division of Pulmonary and Critical Care Medicine, Department of Medicine, University of Michigan Medical School, Ann Arbor, MI, United States; Department of Clinical Pharmacy, University of Michigan College of Pharmacy, Ann Arbor, MI, United States; Division of Pulmonary and Critical Care Medicine, Department of Medicine, University of Michigan Medical School, Ann Arbor, MI, United States; Department of Microbiology and Immunology, University of Michigan Medical School, Ann Arbor, MI, United States; Department of Medicine, University of Virginia School of Medicine, Charlottesville, VA, United States; Division of Pulmonary and Critical Care Medicine, Department of Medicine, University of Michigan Medical School, Ann Arbor, MI, United States; Department of Microbiology and Immunology, University of Michigan Medical School, Ann Arbor, MI, United States; Department of Medicine, University of Massachusetts (UMass) Chan Medical School/UMass Memorial Health System, Worcester, MA, United States; Department of Biostatistics, University of Michigan School of Public Health, Ann Arbor, MI, United States; Department of Medicine, University of Virginia School of Medicine, Charlottesville, VA, United States; Division of Pulmonary and Critical Care Medicine, Department of Medicine, University of Michigan Medical School, Ann Arbor, MI, United States; Department of Microbiology and Immunology, University of Michigan Medical School, Ann Arbor, MI, United States

**Keywords:** gut microbiota, idiopathic pulmonary fibrosis, antibiotics, antifibrotics

## Abstract

**Rationale:**

Gut microbiota modify immunity. Dysregulated immunity plays a key role in the pathogenesis of idiopathic pulmonary fibrosis (IPF). However, the role of gut microbiota in IPF pathogenesis is unknown.

**Objectives:**

Determine associations between gut microbiota, disease severity, and lung transplant-free survival in IPF.

**Methods:**

Gut microbiota from patients enrolled in the CleanUP-IPF trial were characterized using fecal swab samples (*n* = 411). CleanUP-IPF investigated the clinical efficacy of long-term antimicrobials in IPF. 16S ribosomal RNA gene amplicon sequencing and shotgun metagenomic sequencing were performed to comprehensively profile gut microbial communities. Associations between baseline microbiota with disease severity, transplant-free survival, and treatment heterogeneity were analyzed using principal component analysis, multivariate generalized linear models, additive models, and Cox regression models.

**Measurements and Main Results:**

Gut microbiota composition varied significantly with sex, age, and proton pump inhibitor use. Gut microbial diversity and community composition were significantly associated with impaired gas exchange (percent predicted diffusing capacity of lung for carbon monoxide). Several genera including the *Lachnospiraceae unclassified* genus were associated with improved transplant-free survival (hazard ratio [HR] = 0.34; 95% CI, 0.14-0.87; *P* = .02) in patients not assigned to antimicrobial treatment. Patients with a higher abundance of the *Lachnospiraceae unclassified* genus exposed to long-term cotrimoxazole had worse survival (HR = 6.09; 95% CI, 1.36-27.27; *P* = .02). Survival in pirfenidone-treated patients was significantly associated with a higher abundance of the gut *Lachnospiraceae unclassified* genus.

**Conclusions:**

In exploratory post hoc analysis, gut microbiota correlated with disease severity, associated with treatment heterogeneity and transplant-free survival in patients with IPF.

At a Glance Commentary **Scientific Knowledge on the Subject:** Perturbations to gut microbiome have been shown to associate with severity and modify treatment responses in chronic diseases but has been understudied in idiopathic pulmonary fibrosis (IPF).

**What This Study Adds to the Field:** Community composition of the gut microbiome correlates with disease severity in IPF, and higher abundance of certain bacteria at the genus level associates with transplant-free survival and interacts with long-term antimicrobial exposure. These findings suggest the gut microbiome may be an indicator of underlying disease severity and guide further mechanistic and translational work.

## Introduction

The gut microbiome is a key regulator of systemic immunity and implicated in the pathogenesis of myriad diseases.^1^^,^^2^ Dysregulated immunity plays a role in the development of respiratory tract ­diseases,^3-5^ and recent work has highlighted potential casual contributions of respiratory tract microbiota to lung disease.^6-9^ Idiopathic pulmonary fibrosis (IPF) is a common form of interstitial lung disease that is invariably progressive and fatal.^10^ Recent preclinical work supports potential causal associations with gut microbiota and lung fibrosis whereby alterations in gut microbial communities associate with dysregulated pulmonary immunity and fibrosis.^11-13^ However, our understanding of the gut microbiome’s role in the pathogenesis of respiratory disease is limited.^6^ No large-scale human studies have been performed to determine associations between the gut microbiome and clinical outcomes in IPF.

Gut microbiota can shape immunity^14^^,^^15^ and may predict the onset of chronic lung diseases.^9^ However, specific key mechanisms in pulmonary disease remain poorly explored. Therefore, the “gut-lung axis” and its potential therapeutic implications remain undeveloped in IPF. Gut microbiota modify dietary fiber and can produce short-chain fatty acids (SCFA) such as butyrate, acetate, and propionate that interact with G protein-coupled receptors. These interactions can alter immunological and physiological responses,^16^^,^^17^ promoting hematopoiesis and cellular immunity.^18^^,^^19^ Gut microbiota maintain intestinal barrier integrity, and bacterial translocation to the lung can occur during sepsis and acute respiratory distress syndrome promoting increased mortality.^20^ A recent pragmatic clinical trial investigated the effects of long-term broad spectrum antimicrobial agents on clinical outcomes in patients with IPF (NCT02759120).^21^ Given the impact of antibiotics on gut microbiota,^22^ the high biomass of gut microbial communities, and their association with systemic immunity, further work to elucidate the relationship between key microbiome predictors of treatment response, disease severity, and survival may be insightful in IPF.

In this initial report, we performed a post hoc exploration in CleanUP-IPF trial participants and describe the gut microbiota at baseline. We hypothesized that key features of the gut microbiome associates with disease severity and transplant-free survival (TFS).

## Methods

### Study design

CleanUP-IPF was a NHLBI-funded clinical trial (NCT02759120).^21^^,^^23^ In this multicenter randomized control trial, patients were randomly assigned to usual care, oral doxycycline, or oral cotrimoxazole for the duration of the trial. Fecal swabs were collected from patients at baseline and stored (−80°C) for subsequent 16S ribosomal RNA (rRNA) gene sequencing. Institutional review board approval and written consent by participants were obtained at each study site.

### 16S rRNA gene amplicon sequencing

Fecal swabs were submitted to the University of Michigan Microbiome Core where DNA extractions and library preparations were performed using protocols and primers as previously published.^24^ 16S sequence data were processed and analyzed using mothur Version 1.42.3 according to the Standard Operating Procedure for MiSeq sequence data using a minimum sequence length of 250 V4 basepairs.^25^ A shared community file and a phylotyped (genus-level grouping) file were generated using operational taxonomic units binned at 97% identity and classified using the mothur implementation of the Ribosomal Database Project (RDP) Classifier and the RDP taxonomy training set 14 (Trainset14_032015.rdp), available on the mothur website. Data are accessible under the BioProject ID 1314809 at the NCBI SRA.

### Shotgun metagenomic sequencing

Extracted DNA from representative samples (*n* = 41) were sequenced on the Illumina Novaseq (Advanced Genomics Core, University of Michigan), and analysis of metagenome data are as previously described.^8^ Data are accessible under BioProject ID 13148109.

### Bacterial biomass measures

Droplet digital PCR was performed as previously described on DNA extracted from each fecal swab.^26^

### Statistical analysis

Operational taxonomic units were binned to their appropriate genus, and associations with biological and demographic variables, disease severity, and survival were undertaken at the genus taxonomic classification. For analysis, samples were filtered by a mean relative abundance of > 0.5% and a prevalence of > 10% to generate a cohort prevalent microbiome. Unifold mapping approximation and projection was performed on 16S data using the umap R package. The vegan R package was employed to analyze community composition. Count data were normalized and Hellinger transformed in the vegan package and visualized using principal component analysis (PCA). Permutational multivariate analysis of variance (PERMANOVA) from the vegan package (adonis2 function) was used to perform statistical hypothesis testing with the Euclidean distance metric applying 10 000 permutations by default. Assumptions of homogenous dispersion of data points from the spatial median were assessed using the betadisper function and tested with the permutest function. Covariates in adjusted PERMANOVA models included age, sex, smoking status, study region, and proton pump inhibitor (PPI) use. To assess the direction and strength of associations between clinical variables and community composition variation, environmental fitting was performed using the envit function in the vegan package. For differential abundance analysis examining associations with percent predicted diffusing capacity of lung for carbon monoxide (ppDLCO), ppFVC, and antifibrotic treatments, we used generalized linear models (MaAsLin2)^27^ normalized by total sum scaling and log transformed. In MaAsLin2 models we attempted to balance the rates of type 1 and type 2 error using a higher threshold for discovery with a false-discovery rate (FDR) of < 0.2 given the high dimensionality, sparsity, and interindividual variability of microbiome data. These model results are regarded as hypothesis generating and presented as total sum scale relative abundances, and results from log-transformed abundances are reported in the supplementary appendix. Covariates in the adjusted MaAsLin2 models included age, sex, smoking status, study region, antifibrotic use, and PPI use. Shannon diversity index was calculated using the diversity function in the vegan package in samples across all genera. Linear regression models examining disease severity were adjusted for age, sex, smoking status, study region, PPI use, and antifibrotic use. In survival analysis, we used the Benjamini Hochberg procedure with a cutoff for FDR of < 0.10. For survival analysis, Kaplan–Meier survival curves and Cox regression models were used to examine associations of gut microbiota features with 12-month TFS. Cox regression models were adjusted for age, sex, smoking history, baseline ppFVC and ppDLCO, baseline antifibrotic, and PPI use. Multiplicative interaction was used to determine whether gut microbial features modified the associations between antibiotic exposure with 12-month TFS. Pairwise and groupwise differences in distributions were analyzed using Wilcoxon rank sum test or Kruskal–Wallis with Dunns posttest where appropriate. Data are presented as median with 25th and 75th percentiles with minimum and maximum values displayed. All analyses were carried out in R version 4.4.2. (R Core Team [2020]. R: A language and environment for statistical computing. R Foundation for Statistical Computing, Vienna, Austria. https://www.R-project.org/).

## Results

### Characterization of key features of the gut microbiome in patients with IPF

There were 411 patients with valid baseline gut microbiota assessments for this study (Table 1). The majority were men, had a history of smoking, and self-reported antifibrotic and PPI use. Demographics of patients who did not have valid gut microbiota assessments are shown in Table E1. There was a lower proportion of those with a history of smoking and a higher proportion of those who reported to be using pirfenidone; otherwise, demographics were similar between the 2 groups. Total bacterial biomass from swabs showed sample variation, but all clinical samples were significantly higher in biomass than experimental and procedural controls (Figure E1). We used uniform manifold approximation and projection to ordinate the community composition at trial baseline (Figure 1A) with labeling of the most abundant genus in each clinical sample. We next determined cohort prevalent intestinal microbiota in IPF and reported genera in ranked order of abundance (Figure 1B) and prevalence (Figure E2). We analyzed gut microbiota community composition using PCA. We examined the association between key demographic variables and medications with community composition and microbial diversity. For community composition we used PCA and generated ordinations; for microbial diversity we measured the Shannon diversity index (SDI). Biological sex was associated with significant variation in community composition (PERMANOVA *P* = 9.9 x 10^−5^) (Figure 1C; Table E2) but was not associated with significant differences in diversity (Figure 1D). Age stratified by tertile brackets (low: 50.2-68.4 years; intermediate: 68.5-74.9 years; high: 75.0-90.4 years) was significantly associated with variation in community composition (PERMANOVA *P* = .013) (Figure 1E; Table E2) and positively correlated with SDI (*R* = 0.19, *P* = 9.2 x 10^−5^) (Figure 1F). These differences were driven by an increased diversity in older patients based on analysis of gut diversity among age tertiles (*P* = 7.9 x 10^−4^). There was a significant difference in SDI between the high age tertile and intermediate tertile (*P* = .004) and the high age tertile and low tertile (*P* = 5.0 x 10^−4^) in post hoc testing. Baseline PPI use was significantly associated with community composition (PERMANOVA *P* = 9.9 x 10^−5^) (Figure 1G; Table E2) but not associated with significant differences in diversity (Figure 1H). Self-reported gastroesophageal reflux disease was associated with variation in community composition (PERMANOVA *P* = .013), but this was not maintained after adjustment for covariates (Table E2). Smoking status (none vs ever) was not associated with variation in composition (PERMANOVA *P* = .40) (Figure E3; Table E2) and not significantly associated with differences in microbial diversity (*P* = .36) (Figure E4). Study region was also not associated with variation in community composition (PERMANOVA *P* = .136) (Figure E5; Table E2), however significant within-study region heterogeneity was noted on assessment of β diversity variance (*P* = .029) (Figure 1I; Table E2). Finally, we examined differential abundance of genera across demographics and medication exposure using multivariate adjusted generalized linear models (MaAsLin2) (Figure 1J; Table E3). We then performed shotgun metagenomic sequencing in a pilot study of 41 trial participants at baseline and report the most abundant species in fecal communities (Figure 1K). Overall, we found that key demographics and medications were associated with variation in gut microbial community composition and diversity in IPF.

**Figure 1:**
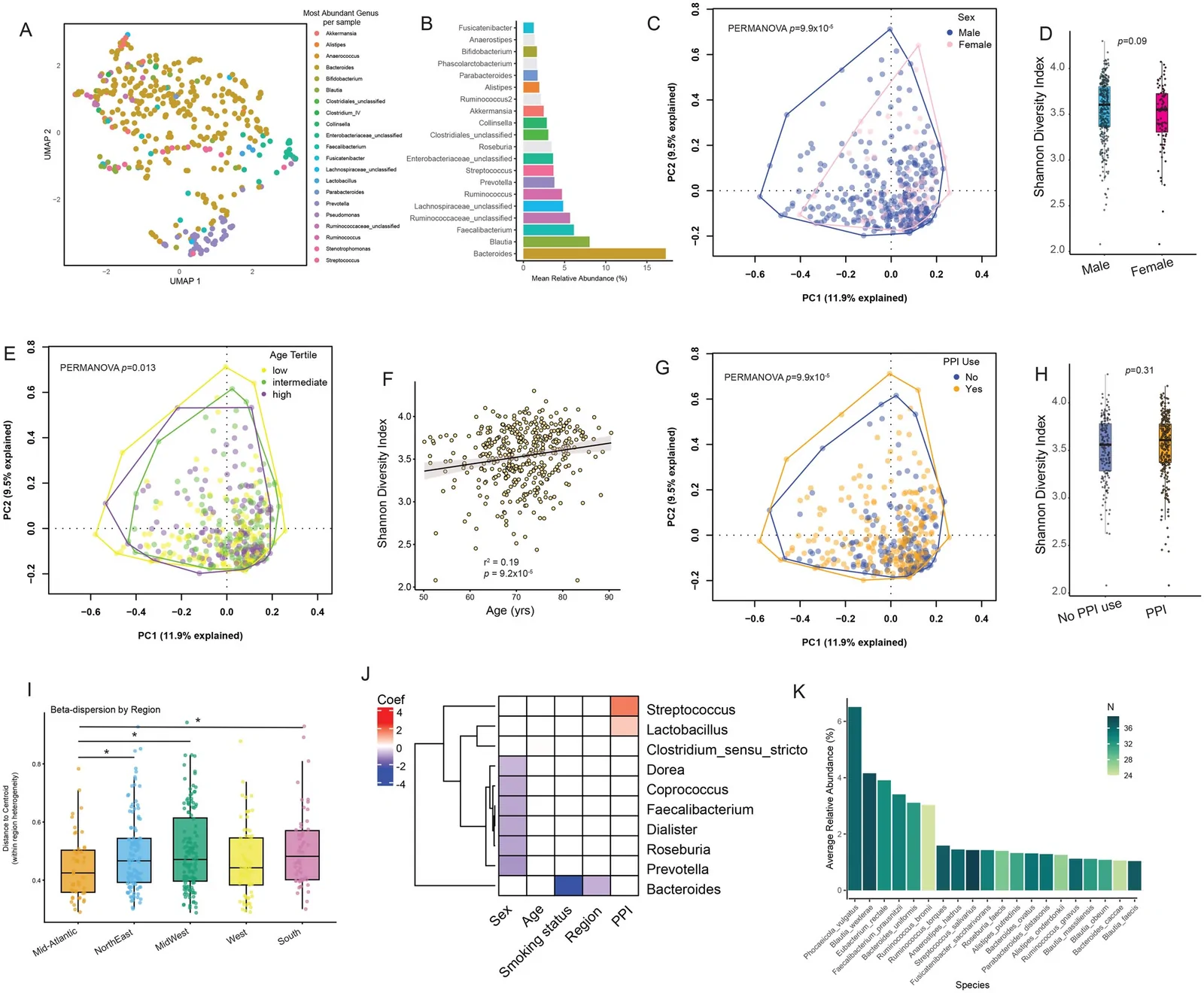
The intestinal microbiota of patients with idiopathic pulmonary fibrosis (IPF) demonstrates variation by key demographic factors and medications. Patients with IPF enrolled in the CleanUP-IPF clinical trial had fecal swabs collected at trial enrollment. Fecal swabs underwent DNA extraction, and gut microbiota were characterized by 16S ribosomal RNA (rRNA) gene sequencing (*n* = 411) and cohort prevalent microbiota examined. (A) Unifold map approximation and projection (UMAP) of gut microbiota from patients at the genus taxonomic classification depicts the community structure with each sample labeled by the most abundant community genus. (B) Community composition reported in order of rank abundance. Grey box depicts a genus that did not reach maximum abundance in any 1 sample. (C) Principal component analysis (PCA) and ordination of community composition with variation by sex. (D) Microbial diversity measured by Shannon diversity index (SDI) in male and female trial participants at baseline. (E) Principal component analysis and ordination of age across low intermediate and high tertiles. (F) Linear regression model of age (x-axis) in years at trial enrollment and SDI (y-axis). (G) Principal component analysis and ordination of community composition in patients on self-reported proton pump inhibitor (PPI) compared to those without PPI use. (H) Shannon diversity index across participants with and without self-reported PPI use. (I) Homogeneity of dispersion (variance form mean/distance from centroid) across geographic study regions. (J) Hypothesis-generating multivariate adjusted generalized linear model (MaAsLin2) of differential abundance across demographic factors and key medications (fixed effects include age, sex, smoking status, study region, PPI use; false-discovery rate [FDR] adjusted *P* = .2). Sex reference: female. Smoking status reference: ex-smoker. Region reference: south. PPI reference: yes baseline use. (K) Shotgun metagenomic sequencing of fecal swabs in pilot study reporting core microbiota (>1% mean abundance, > 50% prevalence) profiled at the species level. Statistical testing (C, E, F): by permutational multivariate analysis of variance (PERMANOVA) with testing for beta diversity variance for each group comparison by permutest, Sex: permutest *P* = .63. Age: permutest *P* = .16. PPI: permutest *P* = .81. (D, H): Wilcoxon rank sum test. (F): univariate linear regression. (I): permutest)

**Figure 2:**
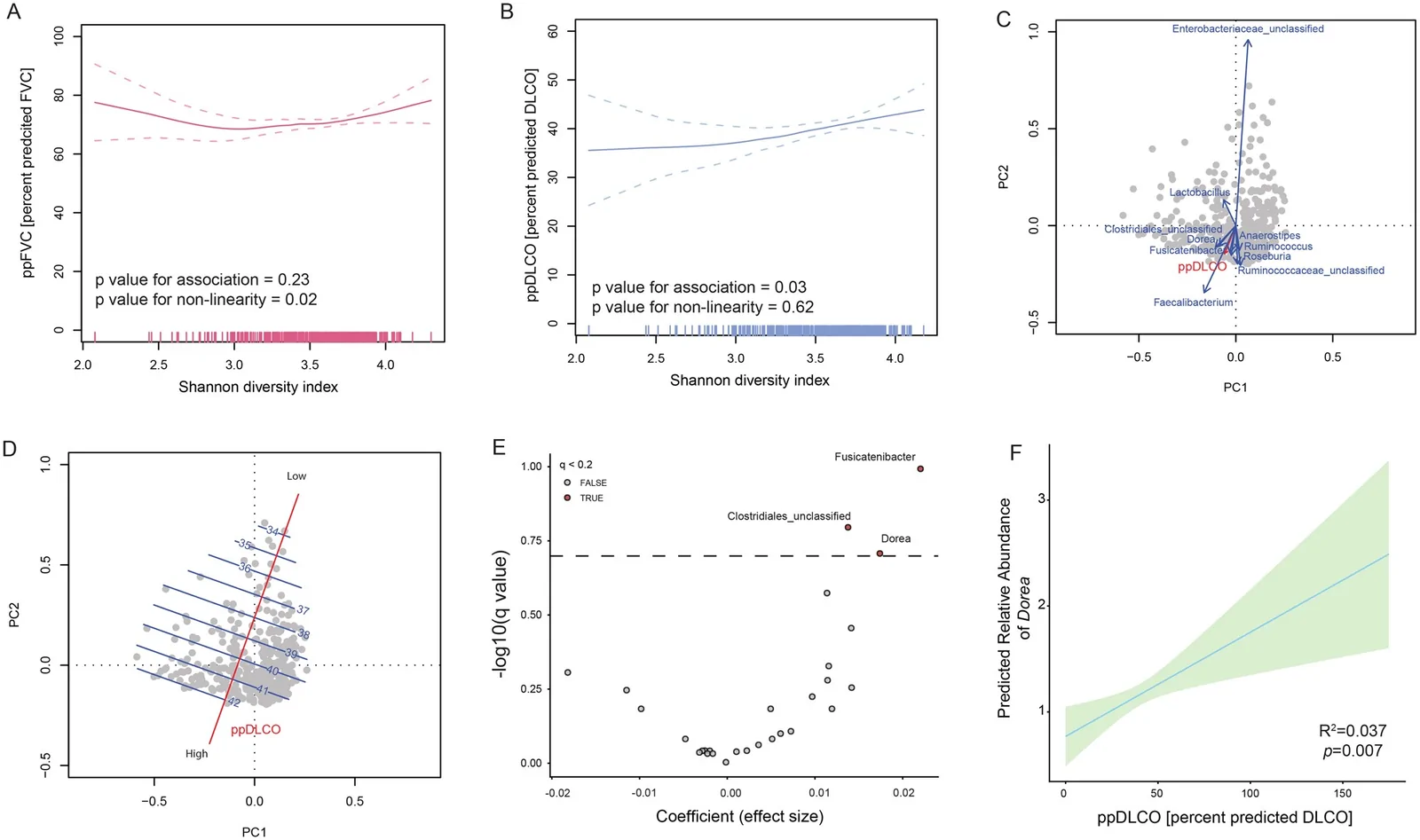
Gut microbiota associate with disease severity in idiopathic pulmonary fibrosis (IPF). Disease severity was assessed using percent predicted diffusing capacity of lung for carbon monoxide (ppDLCO) and ppFVC at baseline and gut microbiota characterized by 16S ribosomal RNA (rRNA) sequencing of fecal swabs at baseline (*n* = 389) prior to trial antibiotic exposure. Cohort prevalent microbiota associations were analyzed. (A) General additive model (GAM) plot of association between Shannon diversity index (SDI) and ppFVC at baseline in the CleanUP-IPF cohort. (B) General additive model plot of association between SDI and ppDLCO at baseline in the CleanUP-IPF cohort. (C) Principal component analysis (PCA), ordination, and biplot depicting post hoc regression of ppDLCO with community composition and the top 10 genera contributing to variation on principal component 2 (PC2) using an environmental fitting function. (D) Diffusing capacity of lung for carbon monoxide gradient plotted across ordination space and aligning with variation on PC2. (E) Hypothesis-generating multivariate generalized linear model (MaAsLin2) fixed effects including age, sex, smoking status, region, PPI use, and antifibrotic use supports associations with *Dorea*, *Fusicatenibacter*, and *Clostridales unclassified* and higher ppDLCO. (F) Multivariate adjusted linear regression model of *Dorea* relative abundance and ppDLCO (adjusted for age, sex, smoking status, PPI, antifibrotic, and region). Statistical testing by (A, B, F): generalized linear model. (C, D): post hoc Spearman correlation.

**Figure 3:**
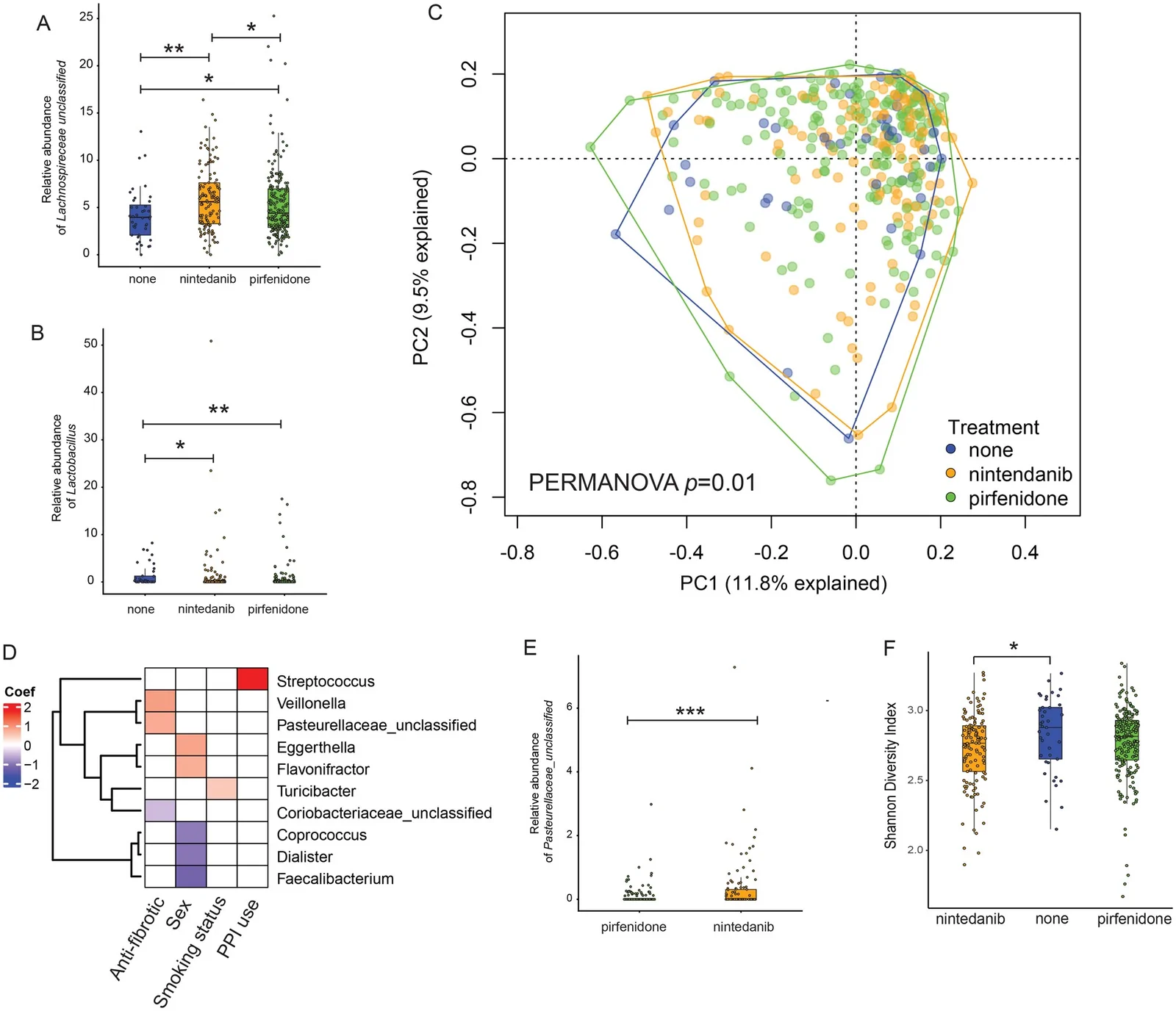
Antifibrotic therapy is associated with gut microbiota perturbation. Patients with idiopathic pulmonary fibrosis (IPF) enrolled in the CleanUP-IPF clinical trial had fecal swabs collected and gut microbiota characterized by 16S ribosomal RNA (rRNA) gene sequencing. Patients were stratified by antifibrotic therapy into 3 cohorts: no antifibrotic therapy (*n* = 41), nintedanib (*n* = 125), or pirfenidone (*n *= 204). (A) Multivariate generalized linear models (MaAsLin2) of cohort prevalent microbiota adjusted for sex, age, smoking status, region, portion pump inhibitor (PPI), and baseline percent predicted FVC (ppFVC) identify the genera *Lachnospiraceae unclassified* and (B) *Lactobacillus* as differentially abundant across treatment groups independent of other fixed effects. (C) Principal component analysis (PCA) and ordination demonstrate significant variation in all gut microbiota (cohort prevalent and rare) supporting structural change across treatment groups. (D) Heatmap of multivariate generalized linear models generated coefficients (MaAsLin2) comparing nintedanib- and pirfenidone-treated trial participants; all genera deployed in model. Adjusted for age, sex, smoking status, region, PPI use, and baseline ppFVC. Features selected for false discovery rate [FDR] value < 0.1. (Antifibrotic reference: nintedanib. Sex reference: female. Smoking status reference: ex-smoker. PPI reference: yes baseline use). (E) Relative abundance of *Pasteurellaceae unclassified* compared in pirfenidone- and nintedanib-treated participants. (F) Alpha diversity measured by Shannon diversity index across all 3 cohorts. Statistical testing: (A, B, F): Kruskal–Wallis rank sum test with post hoc Dunn test and Benjamini Hochberg adjustment. (C): permutational multivariate analysis of variance (PERMANOVA). (E): Wilcoxon rank sum test.

**Figure 4:**
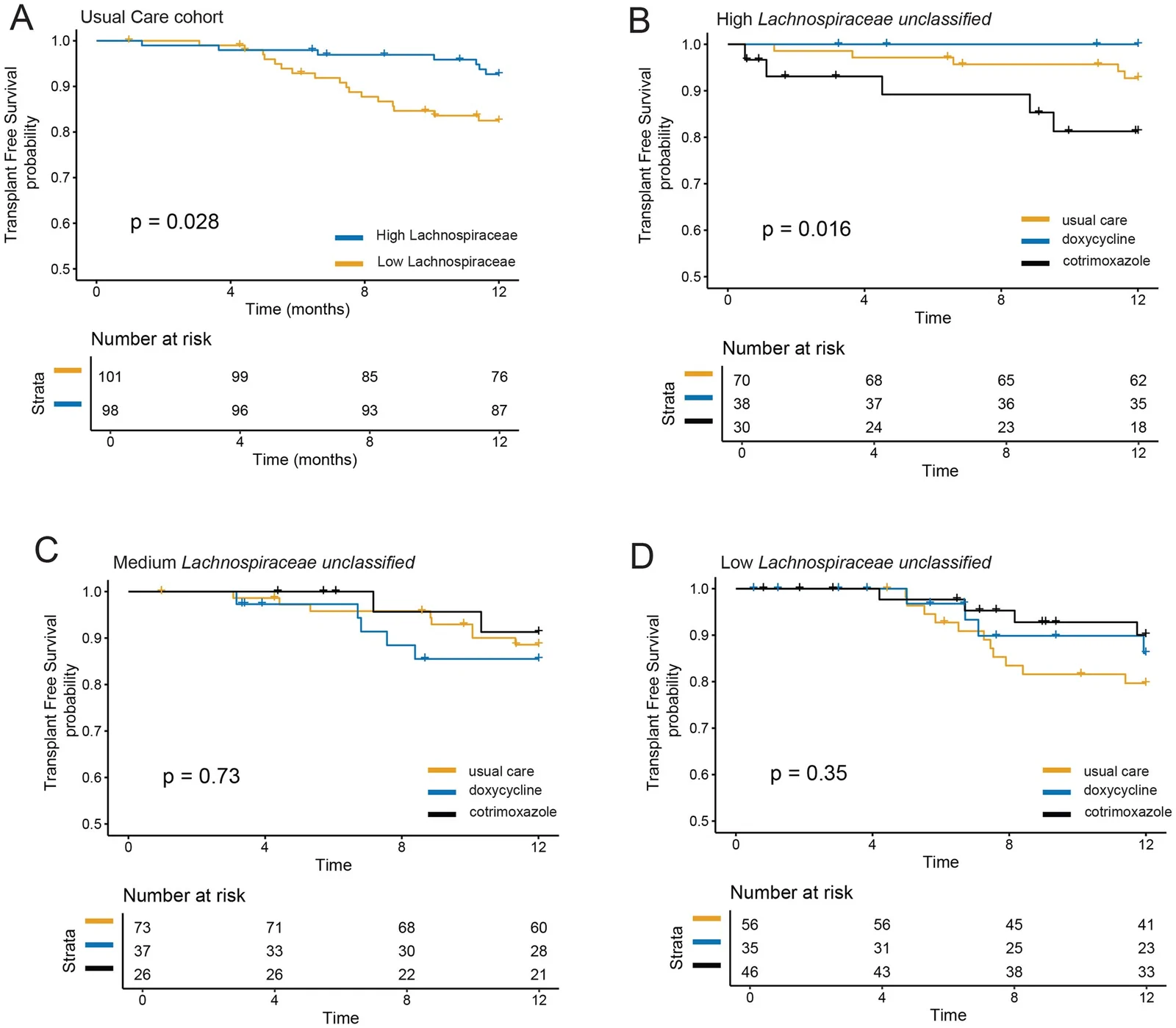
The gut *Lachnospiraceae unclassified* genus in CleanUP-IPF is associated with transplant-free survival, antimicrobial treatment heterogeneity. Patients enrolled in CleanUP-IPF provided fecal swabs at baseline, and gut microbiota were characterized by 16S ribosomal RNA (rRNA) gene sequencing. (A) Kaplan–Meier analysis of transplant-free survival in the usual care cohort (*n* = 199) stratified by above or below median relative abundance of *Lachnospiraceae unclassified*. Patients stratified by *Lachnospiraceae unclassified* genus relative abundance tertiles and Kaplan–Meier analysis across all 3 groups: (B) high tertile, (C) intermediate tertile, and (D) low tertile.

**Table 1:** Baseline characteristics of analyzed cohort.

Characteristics	**Cohort (*n*** = **411)**
**Age, mean (SD), y**	71 (7)
**Female sex, No. (%)**	88 (21.4)
**Smoking history, No. (%)**	270 (65.7)
**White race, No. (%)**	375 (91.2)
**Hispanic ethnicity, No. (%)**	14 (3.4)
**FVC, L, mean (SD)**	2.75 (0.82)
**% predicted FVC, mean (SD)**	71 (18)
**DLCO, mean (SD), mL/min/mmHg**	11.61 (4.60)
**% predicted DLCO, mean (SD)**	40 (16)
**Nintedanib use, No. (%)**	166 (40.5)
**Pirfenidone use, No. (%)**	245 (59.8)
**Proton pump inhibitor use, No. (%)**	248 (60.5)

Abbreviation: DLCO, diffusing capacity of lung for carbon monoxide.

**Table 2:** Baseline characteristics by antifibrotic usage.

Characteristics	**None (*n*** = **41)**	**Nintedanib (*n*** = **125)**	**Pirfenidone (*n*** = **204)**
**Age, mean (SD), y**	73 (6)	71 (7)	71 (7)
**Female sex, No. (%)**	8 (20)	25 (20)	40 (19.6)
**Smoking history, No. (%)**	24 (60)	82 (65.6)	136 (66.7)
**White race, No. (%)**	33 (82.5)	115 (92)	187 (91.7)
**Hispanic ethnicity, No. (%)**	5 (12.5)	6 (4.8)	9 (4.4)
**FVC, L, mean (SD)**	2.94 (0.95)	2.76 (0.79)	2.79 (0.81)
**% predicted FVC, mean (SD)**	77 (21)	71 (17)	71 (18)
**DLCO, mean (SD), mL/min/mmHg**	11.65 (4.10)	11.48 (4.59)	11.91 (4.74)
**% predicted DLCO, mean (SD)**	40 (13)	39 (14)	41 (17)
**Proton pump inhibitor use, No. (%)**	19 (47.5)	71 (56.8)	132 (64.7)

Abbreviation: DLCO, diffusing capacity of lung for carbon monoxide.

**Table 3:** Associations of *Lachnospiraceae unclassified* genus abundance with 12-month transplant-free survival.^a^

Group	Hazard ratio (95% CI)^b^	*P*-value
**Usual care (*n* = 194)**	0.34 (0.14-0.87)	.02
**Doxycycline (*n* = 100)**	0.18 (0.02-1.79)	.14
**Cotrimoxazole (*n *= 95)**	1.89 (0.46-7.75)	.38

a Cox proportional hazards model adjusted for age, sex, smoking history, baseline percent predicted FVC and diffusing capacity of lung for carbon monoxide, antifibrotic use, and proton pump inhibitor use.

b Hazard ratio reported in higher *Lachnospiraceae unclassified* group compared with lower *Lachnospiraceae unclassified* group (reference).

### Gut microbiota features are associated with disease severity in patients with IPF

We next analyzed associations between gut community composition and diversity with lung function. There was no association between ppFVC and SDI (Figure 2A; Table E4). However, higher SDI was associated with a higher ppDLCO (Table E4), and this association was linear (*P* for nonlinearity = .62) (Figure 2B). Shannon diversity index was not associated with TFS (Table E5). We next performed unsupervised PCA and employed an environmental variable fitting function, which generates correlations between selected clinical variables (ppFVC and ppDLCO) with arrows depicting associated gradients in a post hoc approach (Figure 2C). This analysis demonstrated a modest association between ppDLCO and microbial composition (*P* = .042) but not ppFVC (*P* = .87). Key genera contributing to variation on second principal component (PC2) included *Dorea*, *Enterobacteriaceae unclassified*, and *Clostridales unclassified* (Figure 2C) (Table E6). The ppDLCO vector was aligned along the y-axis or PC2, suggesting that variations in ppDLCO correlate with changes in the microbial community structure on this dimension. In addition, we plotted the ppDLCO gradient across PCA space to better understand the association with ppDLCO and PC2 (Figure 2D). We then examined the association between ppDLCO and microbial community structure captured on PC2. In a multivariable linear regression model adjusted for age, sex, smoking status, region, PPI, and antifibrotic use, ppDLCO was modestly associated with summary variation in gut microbiome composition along PC2 (*P* = .01, adjusted *r*^2^ = 0.008) supporting a shift in microbiome composition aligned with impaired gas exchange. We next performed feature selection for cohort prevalent genera and associations with ppDLCO using generalized linear models (MaAsLin2). This model selected several genera associated with higher ppDLCO after adjustment for age, sex, smoking status, region, PPI use, and antifibrotic use (Figure 2E) including *Dorea* (*P* = .008, FDR adjusted *P* = .19), *Fusicatenibacter* (*P* = .002, FDR adjusted *P* = .10), and *Clostridiales unclassified* (*P* = .004, FDR adjusted *P* = .16) (Table E7). However, the effect sizes were modest warranting further replication. *Dorea* and *Fusicatenibacter* are members of the SCFA-producing *Lachnospiraceae* family. The relative abundance by total sum scaling of the *Dorea* genus was positively associated in a multivariable linear regression model with ppDLCO (*r*^2^ = 0.037, *P* = .007) (Figure 2F), supporting it as the strongest candidate for further evaluation. In summary, variation in ppDLCO was associated with microbial α-diversity and modestly associated with changes in community composition at the genus level.

### Antifibrotic therapy associates with perturbation of gut microbiota

Antifibrotic therapy is commonly used in IPF and is associated with adverse gastrointestinal effects and poor tolerability.^28^ We examined the impact of antifibrotic therapy on gut microbial communities at baseline. We stratified our analysis based on self-reported use of nintedanib, pirfenidone, or no antifibrotic therapy at the time of trial enrollment (Table 2). Duration of antifibrotic use prior to study enrollment was not available. We initially examined gut microbial community composition based on cohort prevalent microbiota and found no significant association across antifibrotic groups (PERMANOVA *P* = .28) (Table E8). We next examined cohort prevalent microbiota differential abundance across groups stratified by treatment using multivariate generalized linear models (MaAsLin2) adjusted for sex, age, smoking status, region, PPI use, and baseline ppFVC. We found that within the cohort prevalent microbiome *Lachnospiraceae unclassified* and *Lactobacillus* were differentially abundant across groups ­independent of other fixed effects in the model (Table E9). We report significant differences in the relative abundance of *Lachnospiraceae unclassified* across groups with enrichment in nintedanib groups compared to none (mean nintedanib, 5.91 ± 3.36; mean none group 4.16 ± 2.78; *P* = .003) (Figure 3A) and in pirfenidone compared to none (mean pirfenidone, 5.35 ± 3.77; mean none group, 4.16 ± 2.78; *P* = .03) (Figure 3A). The genus *Lactobacillus* was significantly enriched in none compared to pirfenidone (mean none group, 1.27 ± 2.25; mean pirfenidone 0.59 ± 2.33; *P* = .0023) (Figure 3B) and enriched in nintedanib compared to none (mean nintedanib, 1.38 ± 5.43; mean none group, 1.27 ± 2.25) (Figure 3B). We then hypothesized that antifibrotic exposure may drive changes across less prevalent taxa resulting in subtle compositional shifts and examined all genera detectable in our cohort. We found a significant association between antifibrotic groups and community composition across all genera (global PERMANOVA *P* = .030) (Figure 3C) that maintained significance on adjustment for age, sex, smoking status, region, PPI use, and baseline ppFVC (global PERMANOVA *P* = .010). Within group β-diversity, variance (homogeneity of dispersion) was not significantly different (*P* = .85). Pairwise comparison between nintedanib vs no antifibrotic groups was associated with differences in composition (PERMANOVA *P* = .048), but this association was not maintained after adjustment (PERMANOVA *P* = .051). Pairwise comparisons between pirfenidone and no antifibrotic did not demonstrate a significant association with variation in composition (PERMANOVA *P* = .199, adjusted PERMANOVA *P* = .128). Pairwise comparison of pirfenidone-treated and nintedanib-treated cohorts showed significant changes in composition (PERMANOVA *P* = .036), and significance was maintained after adjustment (PERMANOVA *P* = .015). While ecological structure was not different across cohort prevalent microbiota, antifibrotic therapy has a subtle effect on compositional change across less prevalent genera. We then performed pairwise differential abundance analysis using supervised generalized linear models (MaAslin2) in nintedanib and no antifibrotic groups across all genera. We identified *Porphyromonas* as significantly more abundant in the no antifibrotic group after FDR adjustment and independent of the fixed effects sex, age, smoking status, PPI use, region, and baseline ppFVC (*P* = .0005, FDR adjusted *P* = .012) (Table E10). The relative abundance of *Porphyromonas* was significantly greater in the no antifibrotic group than nintedanib-treated patients (*P* = .005). We examined differentially abundant genera using models in pirfenidone-treated participants compared to no antifibrotic and found *Lactobacillus* genera (*P* = .0003, FDR adjusted *P* = .008) was significantly enriched in no antifibrotic group compared to pirfenidone (Table E11) independent of other fixed effects in the multivariable linear model. Finally, we examined differential abundance across trial participants on nintedanib and pirfenidone (Figure 3D). We report several differentially abundant taxa between groups including *Pasteurellaceae unclassified* (*P* < .00001, FDR adjusted *P* = .002) (Figure 3E), *Veillonella* (*P* = .0002, FDR adjusted *P* = .03), and *Coriobacteriaceae unclassified* (*P* = .001, FDR adjusted *P* = .097) (Table E12). We examined microbial diversity and reported significant variation in SDI across all 3 groups (*P* = .04), with post hoc pairwise testing revealing significant differences between nintedanib and no antifibrotic therapy (nintedanib SDI, SD 2.72 ± 0.26; no antifibrotic SDI, SD 2.82 ± 0.26; *P* = .02). No statistically significant difference was noted in SDI between nintedanib and pirfenidone and between pirfenidone and no antifibrotic therapy (Figure 3E). In summary, analysis supports differential abundance signals in microbiota across treatment groups. Ecological structure is not different across cohort prevalent microbiota by antifibrotic exposure but modestly altered when less prevalent taxa are considered. Differential abundance signatures support that differences in gut microbiota between antifibrotic agents are driven by low abundance and low prevalence taxa.

### Transplant-free survival and gut microbiota in IPF

We next examined associations between survival and gut microbiota. Youden index was used to identify cutoff points of relative abundance for each genus that was associated with TFS in the usual care cohort. After adjustment of log-rank *P*-values for multiple comparisons, we identified 8 genera associated with TFS (Table E13). We then performed multivariate Cox proportional hazards models and found 5 genera that associated with survival (Table E14) including *Dialister*, *Lachnospiraceae unclassified*, *Phascolarctobacterium*, *Clostridium iv*, and *Faecalibacterium.* As an abundant member of the gut microbiome with prior knowledge supporting benefit, we formed a hypothesis-driven approach and examined in further detail the *Lachnospiraceae unclassified* genus. There was no difference in *Lachnospiraceae unclassified* genus relative abundance across all 3 assigned antimicrobial treatment cohorts at baseline (Figure E6). We categorized patients in to high or low *Lachnospiraceae unclassified* groups using the median cutoff value (Figure E7). In the usual care group, the high *Lachnospiraceae unclassified* group had longer survival compared with those who had lower abundance (log-rank *P*  = .028) (Figure 4A). This was also observed in the doxycycline group (Figure E8) but not in the cotrimoxazole group (Figure E9). We found similar associations using multivariable-adjusted Cox regression models (Table 3). We then determined whether antifibrotic type modified the association between *Lachnospiraceae unclassified* and TFS in the usual care (Figures E10-11). In the usual care group, the association of higher *Lachnospiraceae unclassified* with improved TFS was stronger among those who reported using pirfenidone (hazard ratio [HR] = 0.14; 95% CI, 0.03-0.73) compared with nintedanib users (HR = 0.56; 95% CI, 0.09-3.71) (*P* for interaction = .02) (Table E15). This finding was not replicated in the doxycycline or cotrimoxazole groups. Given that associations between *Lachnospiraceae unclassified* and survival were different by treatment assignment, we determined whether there was a significant interaction between *Lachnospiraceae unclassified* and antibiotic exposure. We found that *Lachnospiraceae unclassified* modified the association between long-term antibiotic exposure and 12-month TFS in the CleanUP-IPF trial (*P* for interaction = .02). In patients with higher *Lachnospiraceae unclassified*, the cotrimoxazole group had significantly shorter 12-month TFS (Figure 4B). Conversely, there was no significant difference in survival by antibiotic group among those with medium (Figure 4C) and lower *Lachnospiraceae unclassified* mean relative abundance (Figure 4D). This finding persisted in adjusted Cox regression models (Table E16). Thus, in patients who received usual care or doxycycline, higher mean relative abundance of *Lachnospiraceae unclassified* associated with longer TFS. Notably, patients with higher mean relative abundance of *Lachnospiraceae unclassified* and exposed to cotrimoxazole had much shorter survival, suggesting an interaction between gut microbiome and long-term antibiotic exposure that may confer harm in IPF. While these results support gut microbiome-related predictors of treatment heterogeneity, the results are post hoc and exploratory. Differences in *Lachnospiraceae unclassified* abundance across treatments at baseline further complicate the interpretation of the multiplicative interaction. Shotgun metagenomic sequencing in 41 trial participants at baseline confirmed that the *Lachnospiraceae unclassified* genus consisted of *Agathobacter rectalis* (formerly known as *Eubacterium rectale*) and varied strains of *Lachnospiraceae bacterium* (Table E17).

## Discussion

Gut microbiota correlated with disease severity, and a greater abundance of the *Lachnospiraceae unclassified* genus was associated with longer TFS. In antimicrobial treatment arms of the trial, we report differential survival between doxycycline and cotrimoxazole that associated with gut microbiota variation. Cotrimoxazole-treated patients with greater *Lachnospiraceae unclassified* abundance were subject to reduced survival compared with usual care and doxycycline groups. We also report the same gut microbiota–related signature associated with survival in pirfenidone-treated patients. To our knowledge, our observations are the first in IPF and support further work to identify microbiome-derived biomarkers and potential mechanistic targets.

The gut microbiome produces immunomodulatory metabolites and maintains gut barrier integrity.^29^ Recent preclinical studies implicate the gut microbiome in lung injury and fibrosis suggesting gut microbiota can tailor the immune response to lung injury and wound repair.^11-13^ One of the strongest associations we observed was between *Lachnospiraceae unclassified* and survival. *Lachnospiraceae* is a family of gram-positive obligately anaerobic bacteria that are highly abundant in the human gut microbiome and altered by antimicrobials and changes in diet.^30^^,^^31^ This family is critical to production of SCFAs, secondary bile acids, and lantibiotics (peptide antibiotics) that provide enhanced resistance to gut colonization and modulate host metabolism and immunity.^30^^,^^32-34^ Lower abundance of *Lachnospiraceae* may be detrimental due to impairment of these beneficial pleotropic functions. In this study the *Lachnospiraceae* family consisted of 19 different genera including *Lachnospiraceae unclassified* genus in addition to *Roseburia*, *Blautia*, *Coprococcus*, *Dorea*, and others. 16S rRNA amplicon sequencing has limited taxonomic resolution, and the *Lachnospiraceae unclassified* genus is a functionally diverse genus. Our metagenomic study explored this *Lachnospiraceae unclassified* genus confirming that the genus consisted of various strains of *Agathobacter rectalis* (formerly known as *Eubacterium rectale*) and *Lachnospiraceae bacterium*. Our findings suggest 2 species dominate this genus, but further work is required.

In addition to potential influences on disease progression, the gut microbiome may be an important predictor of the clinical efficacy of pharmacotherapy as gut microbiota can metabolize and modify drugs and their components before target interaction occurs.^35^^,^^36^ Conversely, these interactions can promote enhanced drug efficacy.^37-40^ Studies of immunotherapy and immune checkpoint blockade pioneered these observations and identified molecular gut microbiota–derived targets including candidate *Lachnospiraceae* family members.^41^ Indeed, the inverse associations between *Lachnospiraceae unclassified* abundance and TFS in usual care and doxycycline groups were lost in the cotrimoxazole group. Cotrimoxazole is a commonly prescribed antibiotic with broad antimicrobial activity. Although studies implicated improved survival in IPF with cotrimoxazole,^42^ the CleanUP-IPF and EME-TIPAC trials results did not show a benefit.^21^^,^^43^ Our analysis suggests long-term exposure of cotrimoxazole may have in fact been harmful in a subset of patients with higher *Lachnospiraceae unclassified* in their gut and may partly explain why there was no protective effect in the CleanUP-IPF trial. One reason for this may be that if *Lachnospiraceae* truly has protective effects in IPF, then cotrimoxazole may deplete gut *Lachnospiraceae*. Cotrimoxazole may also have off-target activity that alters secretion of inflammatory cytokines and other immunomodulating mediators, which have been shown in human blood monocytes.^44^ We speculate that cotrimoxazole profoundly alters the host gut microbiome, which leads to reduced production of protective metabolites by microbes that impact immunomodulatory and tissue injury responses in other compartments including the lung. Indeed, other studies have strongly implicated the gut microbiome’s role in regulating immune-mediated diseases that involve other organs as this may be the case for IPF.^15^^,^^45^^,^^46^ Additionally, we report a greater abundance of *Lachnospiraceae unclassified* with antifibrotic treatment compared to no antifibrotic and improved survival in patients with high *Lachnospiraceae unclassified* abundance on pirfenidone. Interpretation of these findings is difficult in the absence of data on adverse side effects and duration of antifibrotic treatment and smaller sample sizes but may indicate a potential relationship between antifibrotic drug efficacy and gut microbiota in IPF. It is also possible that this may be due to type I error and discrepant sample sizes between pirfenidone and nintedanib users as further mechanistic data with repeated and frequent fecal collections and medication adjudication are future research directions. There is a pressing need to improve our understanding of treatment heterogeneity in clinical trials using translational biomarkers including gut microbiome characteristics. Randomized control trials can be leveraged using post hoc translational approaches to identify key traits associated with therapeutic response.

We found that increasing gut microbial diversity correlates with higher ppDLCO and suggests a link between less severe disease and higher gut diversity. Gut microbial diversity has gained attention as urbanized populations display lower microbial diversity, a finding associated with worse inflammatory and chronic disease outcomes.^45^^,^^47^^,^^48^ We found that in IPF, the gut microbiome’s composition associates with DLCO and varies with sex, age, and PPI use. Microbial diversity was correlated with age, and the *Bacteroides* genus was predominant.

There are limitations to this study. Microbiome data are inherently compositional and zero inflated. Observed differences reflect relative shifts in community structure rather than absolute microbial abundance. This constraint can result in spurious correlations and requires cautious interpretation. Our 16S approach has limited taxonomic resolution. While correlations between community composition and disease is promising, the key challenge is determining the functional consequences of this microbiome variation. Our exploratory metagenomic analysis provides data on taxonomy, but future work is required for substantial mechanistic inference. Although our findings persist after accounting for confounders, we cannot rule out residual confounding inherent to a post hoc analysis. This pertains to other influences on the gut microbiome that may also impact IPF, such as diet, supplements, lifestyle choices, and exercise. We did not have monthly fecal sampling over the course of the trial to determine the short-term antibiotic-induced changes in the gut microbiome. Studies that prospectively collect samples every few weeks to months with and without long-term antibiotic exposure will be informative. While we found some differences in microbiome features by antifibrotic use, we did not have data on the duration of antifibrotic use, which may have influenced our findings. Given the cross-sectional nature of this analysis, class imbalance across antifibrotic groups, our limited 16S approach, and the potential impact of gastrointestinal side effects, the implications of this are difficult to interpret and will require further research. Importantly, we cannot conclude a causal association between gut microbiota and lung fibrosis from these investigations given cross-sectional design and absence of several potentially confounding variables. However, the differential survival response to antimicrobial treatment when stratified by key gut microbiota features supports dedicated further work. Our lack of independent replication and healthy volunteer cohorts is considered a major limitation. We did not have independent research cohorts with comprehensive gut microbiome and phenotype data and long-term exposures to antibiotics to replicate our findings, which necessitates further investigation.

We present an initial exploratory study identifying a potential role for the gut microbiome in the pathogenesis of IPF. Further work is required to interrogate candidate taxa, understand their metabolic potential and interactions with key aspects of the immune response, and elaborate further on confounding variables and causality.

## Supplementary Material

aamag249_Supplementary_Data
