# Supplementary material for: Gut microbiota associate with disease severity and survival in idiopathic pulmonary fibrosis
Source: Am J Respir Crit Care Med. 2026 May 19;212(10):2455–66. doi: 10.1093/ajrccm/aamag249 (PMC13624794; doi:10.1093/ajrccm/aamag249)
Supplement: Supplementary file 1 [file aamag249_supplementary_data.zip › Blue-202509-2164OC-R1_Supplement.docx]

**Gut microbiota associates with disease severity and survival in idiopathic pulmonary fibrosis**

John S. Kim MD MS^1*^, Abigail Loe MS^2*^, Shwu-Fan Ma PhD^1^, Piyush Ranjan MS^3^, Jay H. Lipinski BS^3^, Sheridan G. Mikhail^3^, Stephen J. Gurczynski PhD^4^, Xiaofeng Zhou PhD^4^, Gary B. Huffnagle PhD^3,4,5^, John Erb Downward PhD^4^, Joseph D. Metcalf, BS^4^, Nicole Falkowski MS^3^, Kathleen A. Stringer PharmD^3,6^, Robert P. Dickson MD^3,4,^, Yong Huang MD MS^1^, Bethany B. Moore PhD^3,4^, Fernando J. Martinez MD MS^7^, Susan Murray ScD^2^, Imre Noth MD^1*^ and David N. O’Dwyer MB BCh BAO PhD^3,4*^

^1^Department of Medicine, University of Virginia School of Medicine, Charlottesville, VA, USA.

^2^Department of Biostatistics, University of Michigan School of Public Health, Ann Arbor, MI, USA.

^3^Division of Pulmonary and Critical Care Medicine, Department of Medicine, University of Michigan Medical School, Ann Arbor, MI, USA.

^4^Department of Microbiology and Immunology, University of Michigan Medical School, Ann Arbor, MI, USA.

^5^Department of Molecular, Cellular and Developmental Biology, University of Michigan Medical School, Ann Arbor, MI, USA.

^6^Department of Clinical Pharmacy, University of Michigan College of Pharmacy, Ann Arbor, MI, USA.

^7^Department of Medicine, University of Massachusetts (UMass) Chan Medical School/UMass Memorial Health System, Worcester, MA, USA.

^*^Authors contributed equally

**Supplementary Appendix**

**Table E1.** Demographics of CleanUP-IPF participants who did not have valid gut microbiota assessments

| **Characteristic** | **Excluded (102)** |
| --- | --- |
| Age, years | 71 (7) |
| Female sex | 23 (22.6%) |
| Smoking history | 58 (55.9%) |
| White | 100 (98%) |
| Hispanic ethnicity | 2 (2.0%) |
| FVC, liters | 2.73 (0.85) |
| % predicted FVC | 70 (16) |
| DLCO, mL/min/mmHg | 11.66 (4.21) |
| % predicted DLCO | 40 (14) |
| Nintedanib use | 32 (31.4%) |
| Pirfenidone use | 73 (71.6%) |
| Proton pump inhibitor use | 68 (66.7%) |

Abbreviations: DLCO=diffusing capacity of lung for carbon monoxide; FVC=forced vital capacity

Continuous variables presented as mean (standard deviation)

**Table E2.** Adjusted PERMANOVA models of community composition

| **Variable** | **Univariate P-value** | **Multivariate P-value** | **Dispersion P-value** |
| --- | --- | --- | --- |
| Sex | <0.0001 | <0.0001 | 0.630 |
| Age* | 0.013 | 0.004 | 0.166 |
| Smoking status | 0.413 | - | 0.504 |
| Region | 0.136 | 0.102 | 0.048 |
| PPI use | <0.0001 | <0.0001 | 0.808 |
| GERD | 0.013 | 0.559 | 0.890 |
| H2 antagonist | 0.357 | - | 0.150 |
| Anti-microbial | 0.290 | - | 0.677 |

PERMANOVA: permutational multivariate analysis of variance.

*age by tertile brackets (young, intermediate, old)

Anti-microbial: baseline treatment assignment prior to anti-microbial exposure – usual care, doxycycline, cotrimoxazole

Multivariate models adjusted for age, sex, smoking status, region and PPI use where appropriate PPI data missing n=1. GERD data missing=1. PPI: proton pump use at baseline. H2 antagonist: H2 receptor antagonist use at baseline. GERD: gastroesophageal reflux disease

Multivariate models applied when univariate PERMANOVA P less than 0.2

Dispersion P value: tested for homogeneity of multivariate dispersion (i.e. distances to group centroids). Dispersion P by permutest

**Table E3.** Multivariate adjusted of linear modeling results of core microbiota and demographics– MaAsLin2 selected features by q value <0.20

| **feature** | **metadata** | **coef** | **N** | **N.not.0** | **pval** | **qval** |
| --- | --- | --- | --- | --- | --- | --- |
| *Streptococcus* | PPI use | 1.652039 | 411 | 371 | 4.06437205536043e-12 | 1.02422175795083e-09 |
| *Roseburia* | sex | -1.00013 | 411 | 380 | 7.77897861921247e-05 | 0.009802 |
| *Coprococcus* | sex | -0.92261 | 411 | 282 | 0.000195 | 0.016361 |
| *Dialister* | sex | -0.97386 | 411 | 173 | 0.000295 | 0.018605 |
| *Faecalibacterium* | sex | -0.97359 | 411 | 365 | 0.000601 | 0.030297 |
| *Dorea* | sex | -0.80672 | 411 | 335 | 0.000788 | 0.033083 |
| *Clostridium_sensu_stricto* | age | 0.045262 | 411 | 184 | 0.003143 | 0.099696 |
| *Lactobacillus* | PPI use | 0.699643 | 411 | 126 | 0.003165 | 0.099696 |
| *Prevotella* | sex | -1.15733 | 411 | 209 | 0.004482 | 0.125496 |
| *Bacteroides* | smoking status | -2.53296 | 411 | 404 | 0.007425 | 0.170093 |
| *Bacteroides* | region | -0.87257 | 411 | 404 | 0.006937 | 0.170093 |

Abbreviations: proton pump inhibitor=PPI

Results from log_10_-transformed abundances reported in this table.

**Table E4.** Associations of Shannon diversity index with disease severity

| **Group** | **Mean difference in ppFVC (95% CI)** | **P-value** | **Mean difference in ppDLCO (95% CI)** | **P-value** |
| --- | --- | --- | --- | --- |
| Overall cohort | 3.2 (-2.1 to 8.6) | 0.24 | 5.45 (0.83 to 10.08) | 0.02 |

Abbreviations: CI=confidence interval; ppDLCO=percent predicted diffusing capacity of lung for carbon monoxide; ppFVC=percent predicted forced vital capacity

Linear regression model adjusted for age, sex, smoking history, antifibrotic use, and proton pump inhibitor use.

Results reported per 1-unit increment in Shannon diversity index.

**Table E5.** Associations of Shannon diversity index with transplant-free survival

| **Group** | **Hazard ratio (95% CI)** | **P-value** |
| --- | --- | --- |
| Usual care | 2.86 (0.72-11.34) | 0.13 |
| Doxycycline | 0.28 (0.02-4.22) | 0.36 |
| Cotrimoxazole | 1.11 (0.15-8.28) | 0.92 |

Abbreviations: CI=confidence interval

Cox regression model adjusted for age, sex, smoking history, baseline percent predicted forced vital capacity and diffusing capacity of lung for carbon monoxide, antifibrotic use, and proton pump inhibitor use.

Results reported per 1-unit increment in Shannon diversity index

**Table E6**. Top 10 taxa contributing to PC2

| **Taxa** | **PC2** |
| --- | --- |
| *Enterobacteriaceae_unclassified* | 0.9589096 |
| *Faecalibacterium* | -0.3461678 |
| *Roseburia* | -0.2054588 |
| *Ruminococcaceae_unclassified* | -0.1975271 |
| *Fusicatenibacter* | -0.1530768 |
| *Ruminococcus* | -0.1376537 |
| *Lactobacillus* | 0.1327920 |
| *Anaerostipes* | -0.1267676 |
| *Dorea* | -0.1119636 |
| *Clostridiales_unclassified* | -0.1032070 |

**Table E7.** Multivariate linear modeling results of core microbiota and ppDLCO– selected features by q value <0.20

| **feature** | **metadata** | **coef** | **N** | **N.not.0** | **pval** | **qval** |
| --- | --- | --- | --- | --- | --- | --- |
| *Streptococcus* | PPI use | 1.733972 | 411 | 371 | 2.1642778192634e-12 | 7.27197347272504e-10 |
| *Coprococcus* | sex | -1.08815 | 411 | 282 | 2.3886499034508e-05 | 0.002675 |
| *Roseburia* | sex | -1.12931 | 411 | 380 | 2.0273555273267e-05 | 0.002675 |
| *Dorea* | sex | -0.96692 | 411 | 335 | 0.000117 | 0.007895 |
| *Faecalibacterium* | sex | -1.15745 | 411 | 365 | 0.000107 | 0.007895 |
| *Dialister* | sex | -0.94223 | 411 | 173 | 0.000893 | 0.049981 |
| *Lactobacillus* | PPI use | 0.780987 | 411 | 126 | 0.001089 | 0.052251 |
| *Fusicatenibacter* | ppDLCO | 0.022035 | 411 | 322 | 0.00242 | 0.101658 |
| *Clostridiales_unclassified* | ppDLCO | 0.01377 | 411 | 402 | 0.004638 | 0.16009 |
| *Clostridium_sensu_stricto* | age | 0.043941 | 411 | 184 | 0.005152 | 0.16009 |
| *Lachnospiraceae_unclassified* | AF use | -0.72789 | 411 | 407 | 0.005241 | 0.16009 |
| *Clostridiales_unclassified* | AF use | -0.75898 | 411 | 402 | 0.005736 | 0.160602 |
| *Erysipelotrichaceae_unclassified* | sex | -0.72651 | 411 | 268 | 0.007568 | 0.181628 |
| *Prevotella* | sex | -1.14968 | 411 | 209 | 0.007227 | 0.181628 |
| *Dorea* | ppDLCO | 0.017397 | 411 | 335 | 0.008757 | 0.196149 |

Abbreviations: AF=antifibrotic, ppDLCO=percent predicted diffusing capacity for carbon monoxide, PPI=proton pump inhibitor

Results from log_10_-transformed abundances reported in this table.

**Table E8.** PERMANOVA results of anti-fibrotic community composition comparison

| **Variable** | **Genera** | **Univariate P-value** | **Multivariate P-value** | **Dispersion P-value** |
| --- | --- | --- | --- | --- |
| Anti-fibrotic | core | 0.285 | 0.108 | 0.871 |
| Anti-fibrotic | all | 0.035 | 0.010 | 0.596 |

PERMANOVA: permutational multivariate analysis of variance.

Multivariate models adjusted for age, sex, smoking status, region, PPI use and baseline ppFVC.. PPI: proton pump use at baseline.

Dispersion P value: tested for homogeneity of multivariate dispersion (i.e. distances to group centroids). Dispersion P by permutest

**Table E9.** Multivariate linear modeling results of Nintedanib versus no anti-fibrotic versus Pirfenidone – selected features by q value <0.10

| **feature** | **metadata** | **Reference** | **coef** | **N** | **N.not.0** | **pval** | **qval** |
| --- | --- | --- | --- | --- | --- | --- | --- |
| *Streptococcus* | PPI use | yes | 1.773844 | 369 | 331 | 1.04804979028828e-11 | 3.52144729536862e-09 |
| *Faecalibacterium* | sex | female | -1.28093 | 369 | 329 | 3.62750444388674e-05 | 0.006094 |
| *Lactobacillus* | AF use | pirfenidone | -1.51183 | 369 | 114 | 9.11748599176251e-05 | 0.010212 |
| *Coprococcus* | sex | female | -1.00538 | 369 | 252 | 0.000218 | 0.018306 |
| *Dialister* | sex | female | -1.06537 | 369 | 155 | 0.000316 | 0.021208 |
| *Roseburia* | sex | female | -1.00219 | 369 | 341 | 0.000425 | 0.023805 |
| *Lachnospiraceae_unclassified* | AF use | nintedanib | 0.64519 | 369 | 366 | 0.001427 | 0.068511 |

Abbreviations: AF=antifibrotic, ppDLCO=percent predicted diffusing capacity for carbon monoxide, PPI=proton pump inhibitor

Results from log_10_-transformed abundances reported in this table.

**Table E10.** Multivariate linear modeling results of Nintedanib versus no anti-fibrotic – selected features by q value <0.10

| **feature** | **metadata** | **Reference** | **coef** | **N** | **N.not.0** | **pval** | **qval** |
| --- | --- | --- | --- | --- | --- | --- | --- |
| *Eggerthella* | sex | female | 1.224142 | 158 | 66 | 1.82806636710007e-05 | 0.008764 |
| *Streptococcus* | PPI use | yes | 1.57316 | 158 | 142 | 2.43430944632549e-05 | 0.008764 |
| *Porphyromonas* | Nintedanib use | none | 0.825982 | 158 | 18 | 5.24050807628854e-05 | 0.012577 |
| *Dorea* | sex | female | -1.4454 | 158 | 132 | 0.000146 | 0.026349 |
| *Catenibacterium* | age | age | -0.03265 | 158 | 17 | 0.000357 | 0.042796 |
| *Coprococcus* | sex | female | -1.49304 | 158 | 103 | 0.000335 | 0.042796 |

Abbreviations: PPI=proton pump inhibitor

Results from log_10_-transformed abundances reported in this table.

**Table E11.** Multivariate linear modeling results of Pirfenidone versus no anti-fibrotic – selected features by q value <0.10

| **feature** | **metadata** | **Reference** | **coef** | **stderr** | **N** | **N.not.0** | **pval** | **qval** |
| --- | --- | --- | --- | --- | --- | --- | --- | --- |
| *Streptococcus* | PPI use | yes | 1.926053 | 0.314976 | 239 | 216 | 4.17872250333519e-09 | 3.49341201278822e-06 |
| *Flavonifractor* | sex | female | 0.779731 | 0.175937 | 239 | 75 | 1.45420297010956e-05 | 0.006079 |
| *Lactobacillus* | Pirfenidone use | none | 1.490189 | 0.349489 | 239 | 70 | 2.94772220413141e-05 | 0.008214 |

Abbreviations: PPI=proton pump inhibitor

Results from log_10_-transformed abundances reported in this table.

**Table E12.** Multivariate linear modeling results of Pirfenidone versus Nintedanib – selected features by q value <0.10

| **feature** | **metadata** | **Reference** | **stderr** | **N** | **N.not.0** | **pval** | **qval** |
| --- | --- | --- | --- | --- | --- | --- | --- |
| *Streptococcus* | PPI use | yes | 0.27242 | 317 | 282 | 6.0997602334253e-10 | 4.83101010487284e-07 |
| *Flavonifractor* | sex | female | 0.159378 | 317 | 101 | 7.4000799830032e-06 | 0.002891 |
| *Pasteurellaceae_unclassified* | AF use | nintedanib | 0.166951 | 317 | 77 | 1.09506492410019e-05 | 0.002891 |
| *Eggerthella* | sex | female | 0.186265 | 317 | 133 | 1.49357140461591e-05 | 0.002957 |
| *Faecalibacterium* | sex | female | 0.322489 | 317 | 283 | 0.000194 | 0.030682 |
| *Veillonella* | AF use | nintedanib | 0.230163 | 317 | 112 | 0.000259 | 0.034141 |
| *Coprococcus* | sex | female | 0.288548 | 317 | 215 | 0.000641 | 0.070018 |
| *Turicibacter* | Smoking status | Ex-smoker | 0.140128 | 317 | 66 | 0.000707 | 0.070018 |
| *Dialister* | sex | female | 0.313235 | 317 | 132 | 0.000913 | 0.080337 |
| *Coriobacteriaceae_unclassified* | AF use | nintedanib | 0.153581 | 317 | 91 | 0.001226 | 0.097087 |

Abbreviations: AF=antifibrotic, PPI=proton pump inhibitor

Results from log_10_-transformed abundances reported in this table.

**Table E13.** Associations of cohort prevalent genera with 12-month transplant-free survival

| **Genus** | **Log-rank p value** | **Univariate FDR adjusted p value** | **Youden’s point (Relative Abundance)** | **Mean Relative Abundance** |
| --- | --- | --- | --- | --- |
| ***Dialister*** | 0.005182 | 0.068956 | 0.797568 | 0.598386 |
| ***Fusicatenibacter*** | 0.008302 | 0.068956 | 0.218178 | 1.368195 |
| ***Phascolarctobacterium*** | 0.008845 | 0.068956 | 0.18046 | 1.503874 |
| ***Lachnospiraceae_unclassified*** | 0.010216 | 0.068956 | 4.890007 | 4.937715 |
| ***Enterobacteriaceae_unclassified*** | 0.013096 | 0.070717 | 7.943559 | 3.340615 |
| ***Faecalibacterium*** | 0.01792 | 0.08064 | 3.567893 | 6.463374 |
| ***Clostridium_iv*** | 0.025206 | 0.091002 | 0.66419 | 0.921669 |
| ***Anaerostipes*** | 0.026964 | 0.091002 | 0.674639 | 1.40911 |
| ***Clostridiales_unclassified*** | 0.039094 | 0.111689 | 2.972053 | 2.830502 |
| ***Blautia*** | 0.041366 | 0.111689 | 6.565009 | 8.291138 |
| ***Ruminococcaceae_unclassified*** | 0.053592 | 0.11269 | 8.571309 | 5.23729 |
| ***Lactobacillus*** | 0.053734 | 0.11269 | 1.079222 | 1.094459 |
| ***Collinsella*** | 0.054258 | 0.11269 | 4.023152 | 2.770156 |
| ***Alistipes*** | 0.06382 | 0.123081 | 0.132246 | 1.633412 |
| ***Coprococcus*** | 0.080387 | 0.137728 | 1.282252 | 0.61726 |
| ***Clostridium_xl_va*** | 0.085489 | 0.137728 | 0.326176 | 1.118202 |
| ***Streptococcus*** | 0.088953 | 0.137728 | 1.976445 | 3.867666 |
| ***Ruminococcus2*** | 0.091819 | 0.137728 | 3.027381 | 2.008509 |
| *Prevotella* | 0.121354 | 0.172451 | 0.434678 | 3.738344 |
| *Akkermansia* | 0.150646 | 0.203372 | 0.237954 | 2.532961 |
| *Bifidobacterium* | 0.208547 | 0.258234 | 3.545713 | 2.003023 |
| *Bacteroides* | 0.215539 | 0.258234 | 21.38644 | 17.27457 |
| *Clostridium_sensu_stricto* | 0.219977 | 0.258234 | 0.286205 | 0.527435 |
| *Dorea* | 0.240648 | 0.270729 | 1.569324 | 1.056006 |
| *Roseburia* | 0.251414 | 0.271527 | 0.780524 | 3.810073 |
| *Parabacteroides* | 0.382377 | 0.397084 | 1.140144 | 1.426227 |
| *Erysipelotrichaceae_unclassified* | 0.553945 | 0.553945 | 1.017181 | 0.665214 |

Abbreviations: FDR=false-discovery rate

**Table E14.** Genera associated with 12-month transplant-free survival in the CleanUP-IPF usual care cohort

| **Genus** | **HR (95% CI)** | **Adjusted p-value*** |
| --- | --- | --- |
| *Anaerostipes* | 0.47 (0.20-1.41) | 0.11 |
| *Dialister* | 0.08 (0.02-0.61) | **0.03** |
| *Enterobacteriaceae_unclassified* | 1.86 (0.68-5.03) | 0.22 |
| *Faecalibacterium* | 5.18 (1.20-22.40) | **0.04** |
| *Fusicatenibacter* | 0.47 (0.20-1.11) | 0.11 |
| *Lachnospiraceae_unclassified* | 0.31 (0.11-0.83) | **0.04** |
| *Phascolarctobacterium* | 4.99 (1.77-14.10) | **0.01** |
| *Clostridium_iv* | 3.34 (1.41-7.89) | **0.02** |

Abbreviations: CI=confidence intervals; HR=hazard ratio

Hazard ratios generated from Cox proportional hazards model adjusted for age, sex, smoking history, baseline percent predicted forced vital capacity and diffusing capacity of lung for carbon monoxide, antifibrotic use, and proton pump inhibitor use. Analysis performed on all of the genus after filtering and applied Benjamini-Hochberg adjusted p-value with cutoff <0.10.

**Table E15**. Association of Pirfenidone and high *Lachnospiraceae unclassified* abundance with transplant free survival

|  | **Usual care** | | **Doxycycline** | | **Cotrimoxazole** | |
| --- | --- | --- | --- | --- | --- | --- |
| **Stratified – Usual Care** | **HR (95% CI)** | **P-value** | **HR (95% CI)** | **P-value** | **HR (95% CI)** | **P-value** |
| Nintedanib |  |  |  |  |  |  |
| Low *Lachnospiraceae unclassified* | 1.0 (REF) | -- | 1.0 (REF) | -- | 1.0 (REF) | -- |
| High *Lachnospiraceae unclassified* | 0.56 (0.09-3.71) | 0.55 | 0.39 (0.07-1.99) | 0.26 | 0.65 (0.30-1.44) | 0.29 |
| Pirfenidone |  |  |  |  |  |  |
| Low *Lachnospiraceae unclassified* | 1.0 (REF) | -- | 1.0 (REF) | -- | 1.0 (REF) | -- |
| High *Lachnospiraceae unclassified* | 0.14 (0.03-0.73) | 0.02 | <0.01 (<0.01 - >99.99 | 0.97 | 0.09 (0.003-3.36) | 0.19 |

Abbreviations: CI=confidence interval; HR=hazard ratio

Cox proportional hazards model adjusted for age, sex, smoking history, baseline percent predicted forced vital capacity and diffusing capacity of lung for carbon monoxide, and proton pump inhibitor use.

P-values for antifibrotic interaction = 0.02 (usual care), 0.99 (doxycycline), 0.11 (cotrimoxazole)

Hazard ratio reported among those with high *Lachnospiraceae unclassified* (ref. low *Lachnospiraceae unclassified*).

**Table E16.** Associations of *Lachnospiraceae_unclassified* genus abundance with 12-month transplant-free survival

|  | *Lower Lachnospiraceae unclassified* | | *Medium Lachnospiraceae unclassified* | | *Higher Lachnospiraceae unclassified* | |
| --- | --- | --- | --- | --- | --- | --- |
| **Group** | **HR (95% CI)** | **P-value** | **HR (95% CI)** | **P-value** | **HR (95% CI)** | **P-value** |
| Usual care | 1.0 (REF) | -- | 1.0 (REF) | -- | 1.0 (REF) | -- |
| Doxycycline | 0.53 (0.13-2.13) | 0.37 | 0.78 (0.21-2.93) | 0.71 | <0.01 (<0.01 to >99.99) | 0.99 |
| Co-trimoxazole | 0.33 (0.08-1.28) | 0.11 | 0.64 (0.12-3.35) | 0.59 | 6.09 (1.36-27.27) | 0.02 |

Abbreviations: CI=confidence interval; HR=hazard ratio

Cox proportional hazards model adjusted for age, sex, smoking history, baseline percent predicted forced vital capacity and diffusing capacity of lung for carbon monoxide, antifibrotic use, and proton pump inhibitor use.

P-value for interaction = 0.02

**Table E17.** Species and Strain profiling of the *Lachnospiraceae_unclassified* genus by shotgun metagenomic sequencing (n=41)

| **Species/Strain** | **Strain** | **Mean Abundance** |
| --- | --- | --- |
| *Eubacterium_rectale* | SGB4933 | 4.00337675 |
| *Lachnospiraceae_bacterium_2_1_46FAA* | SGB4549 | 0.000128293 |
| *Lachnospiraceae_bacterium_AM48_27BH* | SGB4706 | 0.017896585 |
| *Lachnospiraceae_bacterium_CLA_AA_H215* | SGB4777 | 0.00962575 |
| *Lachnospiraceae_bacterium_CLA_AA_H244* | SGB4993 | 0.388012439 |
| *Lachnospiraceae_bacterium_EP_SM_12S_S03* | SGB4551 | 0.023901707 |
| *Lachnospiraceae_bacterium_NSJ_143* | SGB109665 | 0.000193659 |
| *Lachnospiraceae_bacterium_OF09_6* | SGB4966 | 0.004282308 |
| *Lachnospiraceae_bacterium_OM04_12BH* | SGB4893 | 0.019939 |
| *Lachnospiraceae_bacterium_oral_taxon_096* | SGB4479 | 0.0002545 |
| *Lachnospiraceae_bacterium* | SGB4530 | 0.003729 |
| *Lachnospiraceae_bacterium* | SGB4781 | 0.057942195 |
| *Lachnospiraceae_bacterium* | SGB4782 | 0.049291951 |
| *Lachnospiraceae_bacterium* | SGB4922 | 0.019496667 |
| *Lachnospiraceae_bacterium* | SGB4953 | 0.015520488 |
| *Lachnospiraceae_bacterium* | SGB4967 | 0.001675641 |
| *Lachnospiraceae_bacterium* | SGB82501 | 4.43902E-05 |
| *Lachnospiraceae_bacterium* | SGB82511 | 0.000341951 |
| *Lachnospiraceae_unclassified_SGB47637* | SGB47637 | 0.000324146 |
| *Lachnospiraceae_unclassified_SGB69346* | SGB69346 | 0.000560732 |

**Figure E1. Total bacterial biomass in CleanUP-IPF fecal swabs at baseline.** Fecal swabs underwent DNA extraction and had 16S gene counts measured using droplet digital PCR (ddPCR). Raw count data is log 10 transformed. Controls include negative template samples.

**Figure E2. Prevalence of gut microbiota in CleanUP-IPF.** The most prevalent genera identify on analysis of gut microbiota in the clinical trial patients at baseline.

**Figure E3. Gut microbiota variation with smoking status.** Principal component analysis of gut microbiota data from patients in CleanUP-IPF stratified by smoking status. Statistical testing by PERMANOVA.

**Figure E4.** Smoking status and Shannon diversity index

**Figure E5.** Study region and microbiome composition

**Figure E6. Baseline relative abundance of the *Lachnospiraceae_unclassified* genus across all 3 treatment assigned cohorts in CleanUP-IPF.** Statistical testing by Kruskal Wallis test.

**Figure E7.** Histogram of *Lachnospiraceae_unclassified* in CleanUP-IPF cohort (n=411). Median relative abundance is 4.27 (Q1-Q3, 2.52-6.43). There are 204 patients with a *Lachnospiraceae_unclassified* below median cutoff and 207 patients above the cutoff. X-axis is relative abundance of *Lachnospiraceae_unclassified.*

**Figure E8.** Kaplan Meier analysis and survival curves of doxycycline treated patients in CleanUP-IPF. Stratified by high versus low *Lachnospiraceae_unclassified* abundance at baseline. Log rank P value

**Figure E9.** Kaplan Meier analysis and survival curves of cotrimoxazole treated patients in CleanUP-IPF. Stratified by high versus low *Lachnospiraceae_unclassified* abundance at baseline. Log rank P value

**Figure E10.** Kaplan Meier analysis and survival curves of pirfenidone treated patients in the usual care cohort in CleanUP-IPF. Stratified by high versus low *Lachnospiraceae_unclassified* abundance at baseline. Log rank P value.

**Figure E11.** Kaplan Meier analysis and survival curves of nintedanib treated patients in the usual care cohort in CleanUP-IPF. Stratified by high versus low *Lachnospiraceae_unclassified* abundance at baseline. Log rank P value.
